# The invariant chain CD74 protein is a cell surface binding partner of TIMP‐1 in breast cancer cells

**DOI:** 10.1002/1878-0261.13436

**Published:** 2023-04-28

**Authors:** Mikkel Høeberg, Julie Boertmann Noer, Mette Vixø Vistesen, Annette Bartels, Esben Matzen Bech, Sune Boris Nygård, Ulrik Lademann, Jan Stenvang, Siqi Liu, Anja Thoe Fuglsang, Nils Brünner, José Manuel Afonso Moreira

**Affiliations:** ^1^ Department of Drug Design and Pharmacology, Faculty of Health and Medical Sciences University of Copenhagen Denmark; ^2^ Transport Biology section, Department of Plant and Environmental Sciences University of Copenhagen Denmark; ^3^ Sino‐Danish Center for Education and Research (SDC) Aarhus University Denmark; ^4^ Beijing Institute of Genomics Chinese Academy of Sciences Beijing China

**Keywords:** breast cancer, cell signaling, invariant chain (CD74), receptor, TIMP‐1 interaction

## Abstract

Tissue inhibitor of metalloproteinases‐1 (TIMP‐1) regulates the proteolytic activity of matrix metalloproteinases (MMPs), playing an important role in the homeostasis of the extracellular matrix. Beyond its well‐known role in tissue maintenance, TIMP‐1 has been associated with multiple MMP‐independent cytokine‐like functions. The protein structure of TIMP‐1, with two distinct domains, one interacting with MMPs and another able to bind multiple partners, provides a rationale for this multifunctionality. The identification of CD63 as a cell surface receptor for TIMP‐1, able to mediate intracellular signaling through the Erk/MAPK axis, provided a molecular basis for the role of TIMP‐1 in cellular signaling. However, several lines of evidence suggest that TIMP‐1 may be able to associate with many interaction partners, thus attaining multiple functions. To enable the identification of previously unknown interaction partners that may underpin the core cellular functions of TIMP‐1, known as well as unknown, we performed a yeast two‐hybrid screening using a mammary gland complementary DNA (cDNA) library. We report here the identification of multiple interactors, including MHC class II‐associated invariant chain γ (CD74). We verified that CD74 interacts with TIMP‐1 in breast cancer cells and that this interaction contributes to cellular internalization of TIMP‐1 and mediates intracellular signaling through the Akt signaling axis in breast cancer cells. These data provide new insights into the complex nature of the functions of TIMP‐1 and their potential mechanistic basis.

## Graphical abstract

We performed an Y2H screening of a mammary gland cDNA library and identified 56 unique potential TIMP‐1 interactors. We verified one of these interactors, CD74, as a TIMP‐1 cell surface binding protein in breast cancer cells. The remaining hits will need to systematically, and experimentally, be confirmed as TIMP‐1 bona fide binders.

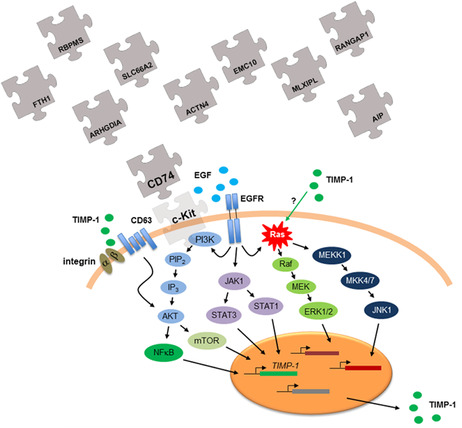

**Abbreviations**
CD74: MHC class II‐associated invariant chain γ
cDNA: complementary DNA
ECM: extracellular matrix
IHC: immunohistochemistry
IP: immunoprecipitation
MAPK: mitogen‐activated protein kinase
MHC: major histocompatibility complex
MIF: macrophage migration inhibitory factor
MMPs: matrix metalloproteinases
MST: microscale thermophoresis
P‐Akt: phospho‐Akt
PBS: phosphate buffered saline
PBS‐T: PBS containing 0.1% Tween 20
PLA: proximity ligation assay
rTIMP‐1: recombinant TIMP‐1 protein
siRNA: small interfering RNA
STR: short tandem repeat
TIMP‐1: tissue inhibitor of metalloproteinases‐1
Y2H: yeast two‐hybrid

## 1 Introduction

Tissue inhibitor of metalloproteinases 1 (TIMP‐1) is one of four known endogenous inhibitors of matrix metalloproteinases (MMPs). By regulating the activity of its cognate MMPs, TIMP‐1 plays an important role in the remodeling of the extracellular matrix (ECM) not only during normal tissue development and homeostasis, but also in tissue pathogenesis [1]. However, the biological functions of TIMP‐1 are not limited to its intrinsic involvement in ECM turnover; in recent years, TIMP‐1 has become increasingly recognized as a multifunctional protein that, independently of its MMP inhibitory activity, is able to regulate various cellular processes such as cell proliferation [2, 3], apoptosis [4], stem cell differentiation [5], and homeostasis of neutrophil granulocytes [6]. Despite abundant evidence directly associating TIMP‐1 with regulation of multiple cellular processes, the mechanisms underlying these effects, and its biological consequences, have remained largely unclear. Only recently have researchers begun to investigate the downstream mechanisms of TIMP‐1–mediated cell signaling [7]. High‐affinity binding of TIMP‐1 to the cell surface of myeloid leukemia cells and keratinocytes has led to the suggestion that TIMP‐1 can signal directly through cell surface receptors [8]. In 2006, CD63, a member of the tetraspanin family of transmembrane proteins, was identified as a cellular binding partner for TIMP‐1 able to regulate cell survival and polarization in MCF10A breast epithelial cells [9]. Subsequently, CD63/integrin β1/TIMP‐1 were shown to form a supramolecular complex with multiple cellular roles (reviewed in ref. [10]). Three additional receptors, Low‐density lipoprotein receptor‐related protein 1 (LRP1) [11], Amyloid Precursor Protein (APP) [12], and CD82 [13], have all been identified as TIMP‐1 interaction partners, lending some support to the hypothesis that TIMP‐1 may have a large interactome able to mediate a broad range of cellular effects [14]. To expand our knowledge on the interactor network of TIMP‐1 and gain some molecular insight into known, as well as unknown functions of TIMP‐1, we performed a yeast two‐hybrid (Y2H) screening with TIMP‐1 as bait using a mammary gland cDNA library. We discovered several potential interactors, and on closer inspection identified MHC class II‐associated invariant chain peptide (CD74), a protein known for its internalization properties, as a TIMP‐1 binding partner in breast cancer. We show here that CD74 is involved in the internalization of TIMP‐1 by breast cancer cells and that TIMP‐1‐mediated activation of Akt signaling is dependent on CD74. To determine the applicability of our findings to a broader context, we analyzed a breast cancer patient cohort for expression of CD74, CD63, and TIMP‐1 by immunohistochemistry (IHC).

## 2 Materials and methods

### 2.1 Yeast cell culture

Yeast cells were grown on YPD Broth (yeast extract peptone dextrose: 1% yeast extract, 2% peptone, and 2% glucose) or on synthetic defined (SD) medium containing 2% D(+)‐glucose, 0.67% yeast nitrogen base without amino acids, dropout supplements, and succinic acid. The dropout (DO) medium was made without leucine, tryptophan, histidine, or adenine and is referred to as ‐Leu, −Trp, ‐His, or ‐Ade, respectively.

### 2.2 Mammalian cell culture

Ramos Burkitt lymphoma cells (RRID: CVCL_0597), MDA‐MB‐231 (RRID: CVCL_0062), MCF‐7 (RRID: CVCL_0031), and T47D (RRID: CVCL_0553) human breast cancer cells used in this study were purchased from ATCC (Rockville, MD, USA). Fetal Calf Serum (FCS) was heat‐inactivated before use, and all cell lines were grown at 37 °C in a humidified incubator with 5% CO_2_. MDA‐MB‐231 cells were maintained in DMEM medium supplemented with 10% FCS, whereas Ramos, MCF‐7, and T47D were cultured in RPMI‐1640 Glutamax medium supplemented with 10% and 5% FCS, respectively. All cell culturing reagents were purchased from Life Technologies (Carlsbad, CA, USA). Origin of all cell lines was verified by short tandem repeat (STR) profiling (IdentiCell, Aarhus, Denmark), and all cells were routinely tested for mycoplasma infection (Minerva Biolabs, Berlin, Germany).

### 2.3 Yeast two‐hybrid screening

*TIMP‐1* cDNA lacking the 23 amino‐acid N‐terminal peptide signal sequence was subcloned into the yeast expression vector pAS2 (Invitrogen, Waltham, MA, USA), forming an in‐frame fusion with the GAL4 DNA binding domain (a.a. 1–147). In‐frame fusion and correct orientation of *TIMP‐1* was verified by DNA sequencing. Expression of the fusion protein was furthermore confirmed by western blotting using an anti‐TIMP‐1 antibody (data not shown). Prior to the screening, we did a series of transformation tests to exclude false activation of the reporter genes by the bait protein. For yeast two‐hybrid screening, the yeast strain PJ69‐4A [MATa *trp1*‐Δ901 *leu2*‐3 112 901 *ura3*‐52 *his3*‐Δ200 *gal4*Δ *gal*80Δ GAL2‐ADE2 LYS2::GAL1‐HIS3 met2::GAL7‐lacZ] [15] was cotransformed with the pAS2/*TIMP‐1* vector and an pATC2‐based human mammary gland cDNA library (Human Mammary Gland MATCHMAKER cDNA library, Clontech, Mountain View, CA, USA) by LiAc‐mediated transformation. In brief, 300 mL YPD medium was inoculated with an overnight yeast culture to obtain an OD_600_ of 0.5. The culture was incubated at 30 °C, 200 rpm for 4–5 h until cells completed at least two cell divisions. The cells were harvested by centrifugation at 3000 ***g*** for 5 min and washed in 50 mL of ice‐cold sterile water and centrifuged again using the same conditions. The cells were resuspended in a transformation mix consisting of PEG 4000 50% w/v, LiAc (0.1 m), herring testis carrier DNA (6 mg), 100 μg bait and 50 μg prey plasmid DNA and incubated at 30 °C for 30 min followed by heat shock at 42 °C for 60 min. Cells were then centrifuged at 3000 ***g*** for 5 min, resuspended in sterile water, and plated onto SD/−Leu/−Trp/−Ade/‐His agar plates with 1 mm 3‐amino‐1,2,4‐triazole to eliminate background growth. Plates were incubated at 30 °C for 5–7 days, and plasmids were isolated from growing colonies by mini‐prep method (Sigma‐Aldrich, St. Louis, MO, USA) and subsequently amplified in *E. coli*. Bait and prey plasmids were retransformed into PJ69‐4A to confirm their interaction. Prey plasmids also showing positive interaction with TIMP‐1 in the second round of transformation were sequenced (Eurofins MWG Operon, Ebersberg, Germany) using a Gal4AD primer (5′‐TACCACTACAATGGATG‐3′), and clones identified by sequence comparison using BLAST (*Homo sapiens* Nucleotide BLAST; NCBI, National Library of Medicine).

### 2.4 Reagents

The his6‐tagged human TIMP‐1 protein (rTIMP‐1) used in this study was from our laboratory. Production and validation of the recombinant protein was previously published [16]. The added his6 tag not only provided an easy purification procedure, but also allows distinguishing between exogenously added his6‐rTIMP‐1 and endogenously produced TIMP‐1, as the two proteins differ by 2 kDa (30 and 28 kDa, respectively). Macrophage migration inhibitory factor (MIF) human recombinant protein produced in *E. coli* was purchased from ProSpec Bio (ProSpec‐Tany TechnoGene Ltd. International, Rehovot, Israel).

### 2.5 Immunoprecipitation

The Dynabeads Co‐Immunoprecipitation Kit (Life Technologies) was used for all IP and co‐IP experiments. The experiments were carried out according to the manufacturer's instruction with minor adjustments. In short, MDA‐MB‐231 cells were incubated with rTIMP‐1 (2.5 μg·mL^−1^) for 15 min and subsequently washed three times in ice‐cold PBS. Cells were then lysed in modified Extraction Buffer (1xExtraction Buffer, 100 mm NaCl, 2 mm MgCl_2_, 1 mm DTT and PMSF) for 15 min on ice. Lysates were centrifuged at 2600 ***g*** for 5 min to pellet large cells debris and nuclei. Supernatants were incubated for 1 h at 4 °C with antibody coupled beads (Clone LN‐2, Santa Cruz Biotechnology, TX, USA); VT4 clone, an in‐house antibody [17] and IgG control, (Santa Cruz Biotechnology) for immunoprecipitation of CD74, TIMP‐1 and negative control, respectively. Following a total of four washes in modified extraction buffer and Last Wash Buffer, respectively, the immunoprecipitated proteins were eluted and subjected to SDS/PAGE and western blot analysis.

### 2.6 Western blot analysis

Whole‐cell lysates were obtained by lysis of the cell monolayer with M‐PER Mammalian Protein Extraction Reagent supplemented with protease and phosphatase inhibitors (Thermo Scientific, Waltham, MA, USA). Lysates were centrifuged at 14 000 ***g*** for 10 min to remove cell debris. Protein concentration of collected samples was measured using the BCA protein Assay Kit (Novagen, Merck KGaA, Darmstadt, Germany) according to the manufacturer's instructions. Equal amounts of protein samples in Laemmli Sample Buffer, 50 mm DTT (Bio‐Rad, Hercules, CA, USA), were heated for 10 min at 70 °C and subsequently subjected to reducing SDS/PAGE. After electrophoresis, the proteins were blotted onto PVDF membranes, which were subsequently blocked with 5% milk in phosphate‐buffered saline (PBS) containing 0.1% Tween 20 (PBS‐T) for 1 h at room temperature (RT). The membranes were then incubated overnight with primary antibodies in PBS‐T containing 5% milk at 4 °C. After three washes in PBS‐T, membranes were incubated with the appropriate secondary antibodies. The antigen was detected using the Amersham ECL‐Select Western Blotting detection reagent (GE Healthcare Life Sciences, Piscataway, NJ, USA) or Clarity Western ECL Substrate (Bio‐Rad) according to the manufacturer's instruction and visualized by UVP BioSpectrum Imaging system (Fischer Scientific, Thermo Fisher Scientific). VT‐7 anti‐TIMP‐1 antibody was an in‐house antibody previously described [17]. Polyclonal rabbit anti‐Akt antibody and polyclonal rabbit anti‐P‐Akt (Ser473) antibody were from Cell Signaling Technologies, Danvers, MA, USA. Monoclonal mouse anti‐CD74 (clone LN‐2) antibody was from Santa Cruz Biotechnology, mouse monoclonal anti‐CD63 (clone NKI/C‐3) antibody was from EMD Chemicals, Billerica, MA, USA, and monoclonal mouse anti‐p150^Glued^ antibody was obtained from BD Transduction Laboratories, Hoboken, NJ, USA.

### 2.7 Transient knockdown of CD74 by siRNA

We tested various siRNAs constructs (data not shown), and the most efficient single siRNA (> 90% downregulation) was used for all downregulation experiments described in this study. Efficiency and duration of the knockdown was also evaluated by western blotting. The lowest level of CD74 was detected 72‐h post‐transfection where approximately 5–10% of wild‐type expression remained. Downregulation of CD74 expression remained stable for up to 144‐h post‐transfection (data not shown). CD74 was transiently downregulated with a synthetic siRNA (SASI_Hs01_00142175, Sigma), and the MISSION^®^ siRNA Universal Negative Control #1 from Sigma was used as control. Cells were plated 24 h prior to transfection, and medium was changed 1 h before start of transfection. According to the manufacturer's instruction, siRNA CD74 (50 nm) was transfected into MDA‐MB‐231 cells using Lipofectamine 2000 (Invitrogen).

### 2.8 Uptake studies

Medium was exchanged 72 h after transfection with fresh medium containing 5 μg·mL^−1^ bioactive his6‐tagged recombinant TIMP‐1 protein (rTIMP‐1) expressed in mammalian cells [16]. Cells were seeded to reach a confluence of 70–80% at time of harvest, and samples were collected after incubation with rTIMP‐1. Before cell lysis, cells were washed at least thrice in ample amounts of ice‐cold PBS buffer. Whole‐cell lysates were obtained as described above, and the amount of rTIMP‐1 taken up by the cells was evaluated by western blot analysis.

### 2.9 Signaling assays

MDA‐MB‐231 cells were transfected with control siRNA or CD74‐specific siRNA, as described above. Then, 72‐h post‐transfection, medium was exchanged with fresh medium (10% serum) containing 50 μg·mL^−1^ rTIMP‐1. Cells were seeded to reach a confluence of 70–80% at time of harvest, and samples were collected after 0, 10, and 30 min of incubation with rTIMP‐1. Before cell lysis, cells were washed three times in ice‐cold PBS. Whole‐cell lysates were obtained as described above, and phosphorylation state of Akt was evaluated by western blot analysis.

### 2.10 Chemotaxis assay

Transmigration of Ramos Burkitt's lymphoma cells was assessed in Transwell chemotaxis chambers (Costar cell culture inserts, Sigma Aldrich) using inserts with a pore size of 5 μm. Briefly, cells were suspended in medium (RPMI 1640, 10% FCS), and a total of 1 × 10^6^ cells was loaded in the upper chamber of the transwell culture insert. Inserts were transferred into the wells containing medium in the presence or in the absence of MIF (250 ng·mL^−1^), or TIMP‐1 (5 μg·mL^−1^). The chambers were incubated for 4 h at 37 °C in 5% CO_2_. After incubation, filters were collected and cells that had migrated into the lower chamber counted. Results are presented as a chemotaxis index (CI), with values normalized to spontaneous migration of B cells in medium in the absence of the chemoattractant.

### 2.11 Clinical samples

Human breast cancer tissue samples were provided by the Department of Pathology of the Copenhagen University Hospital according to the standards set in the Declaration of Helsinki. The study protocol was approved by the Copenhagen and Frederiksberg regional division of the Danish National Committee on Biomedical Research Ethics (protocol nr. H‐3‐2010‐116), and granted exemption from obtaining written informed consent (as per section 10, subsection 1, of the Committee Act). We collected 43 formalin‐fixed, paraffin embedded (FFPE) tissue blocks from patients that underwent mastectomy between 2003 and 2008. The samples included estrogen‐receptor positive and negative tumors as well as different tumor sizes and malignancy grades.

### 2.12 Immunohistochemistry

Freshly cut breast cancer tissue sections or paraffin embedded cell culture sections were deparaffinized and rehydrated through a graded series of ethanol. Antigen retrieval was carried out by boiling the sections for 10 min in 10 mm citrate buffer pH 6.0, followed by a cooling period of 30 min at RT. Sections were blocked with 1% hydrogen peroxide for 10 min prior to incubation with primary antibody, which was carried out overnight at 4 °C. 0.25 μg·mL^−1^ mouse monoclonal antibody VT‐7, diluted in 0.25% BSA in TBS (0.05 m Tris–HCl, 0.15 m NaCl) was used for TIMP‐1 detection. For CD63 detection, 0.5 μg·mL^−1^ mouse monoclonal antibody (Calbiochem/Merck4Biosciences, San Diego, CA, USA) was applied diluted in 0.25% BSA in TBS. For detection of CD74, we used 0.4 μL·mL^−1^ mouse monoclonal antibody LN‐2, diluted in 0.25% BSA in TBS. Binding of primary antibody was detected with Advance HRP (DAKO, Glostrup, Denmark) according to the manufacturer's instructions. All staining procedures were performed manually, and the reactions were visualized by incubating the sections with DAB+ (DAKO) for 5 min. Between incubations, the sections were washed with Tris‐buffered saline (TBS), pH 7.6 containing 0.5% Triton X‐100. The sections were counterstained with Mayer's hematoxylin. We utilized the H‐scoring system described by Ishibashi and colleagues [18] for semiquantitative analysis of the 43 immune‐stained breast cancer tissue samples used in this study. In short, an H‐score was generated by adding the percentage of weakly stained cells (×1), the percentage of moderately stained cells (×2), and the percentage of strongly stained cells (×3), resulting in a possible range of 0–300. The score was evaluated independently by two authors (AB and SBN). As a negative staining control, 2 μg·mL^−1^ IgG_1_ isotype control (DAKO) was used as primary antibody.

### 2.13 Proximity ligation assay

Detection of TIMP‐1 and CD74 protein–protein interaction was investigated using Duolink *In Situ* (OLINK Bioscience, Uppsala, Sweden) according to the manufacturer's instructions. Images were taken using a LSM710 confocal laser scanning microscope with a Plan‐Apochromat 63×/1.40 oil objective and ZEN software (Carl Zeiss ZEN Version 8.0, ZEISS Microscopy, Jena, Germany). Multiple *z*‐stack sections were captured with sections spanning entire cells. Quantification of PLA signals (red puncta) was performed on discrete regions on maximum projections of cross‐sections. The number of puncta per cell was counted with the imagej software [19] and normalized to the number of cells counted to obtain mean PLA puncta/cell values. The microscope settings were kept constant for all images to enable direct comparison. Significance was evaluated with ANOVA, using a significance level of 0.05.

### 2.14 Statistical analysis

Data were analyzed using graphpad prism 9.0 (GraphPad Software, San Diego, CA, USA).

## 3 Results

### 3.1 Yeast two‐hybrid screening identified multiple TIMP‐1 interacting proteins

To identify novel TIMP‐1 binding partners that could give some insight into known, as well as unknown, functions of TIMP‐1, we performed a yeast two‐hybrid (Y2H) screening with a human mammary gland cDNA library. As bait, we used a *TIMP‐1* cDNA construct lacking the 23aa N‐terminal signal peptide domain. Approximately 6.7 × 10^6^ independent transformants were screened on selective SD plates. This initial screening resulted in 133 independent colonies able to grow on selective plates. Plasmids were isolated from all 133 colonies and reintroduced into *E. coli* for amplification and purification. Restriction enzyme analysis was used to identify those plasmids that carried a genuine prey insert. Subsequently, each purified prey plasmid was cotransformed individually with the *TIMP‐1* containing bait plasmid and plated on selective SD plates to confirm that the interaction was caused by the isolated clone. This second test revealed 78 true positive clones, which were sequenced and identified with BLAST sequence analysis, resulting in the discovery of a total of 56 unique TIMP‐1 potential interaction partners (Table 1). Of the 56 potential interactors, two were previously reported TIMP‐1 binding partners—CD63 and LRP1 [9, 11], with one of them, CD63, being a well‐characterized TIMP‐1 receptor. Another hit, c‐Kit, was validated as a TIMP‐1 interactor and reported elsewhere [20]. Strikingly, after we reported in a scientific symposium the invariant chain (CD74)—the very first identified hit in our Y2H screening—as a possible interaction partner for TIMP‐1 [21], our finding was pursued by others and validated in human B lymphoma cells [22], and CD74 predicted to bind the N‐terminal protease inhibitory domain of TIMP‐1. Overall, these data support the validity of our approach and suggest that additional bona fide TIMP‐1 interactors are present among the hits from our Y2H screening, making this a valuable resource for future studies of TIMP‐1 cellular function(s).

**Table 1:** TIMP‐1 interacting proteins found in the Y2H screening.

Interactor	Number of clones	Description	Aliases	Subcellular localization^a^	Gene ontology –biological process^b^
ARHGDIA	4	Rho GDP dissociation inhibitor alpha	RHOGDI, GDIA1, NPHS8	Nucleus, cytosol, cytoskeleton	Regulation of cell adhesion, Rho protein signal transduction
FTH1	4	Ferritin heavy chain 1	FTH, PIG15, FTHL6, PLIF, FHC	Lysosome, nucleus, cytosol, extracellular	Iron ion transport, cellular iron ion homeostasis
CD63	3	CD63 molecule	MLA1, ME491, LAMP‐3, OMA81H, TSPAN30	Lysosome, endosome, extracellular, **plasma membrane**	Regulation of receptor internalization, cell‐matrix adhesion, protein transport
CD74	3	major histocompatibility complex, class II invariant chain	DHLAG, Ia‐GAMMA, HLADG, P33, II, Ii	Golgi apparatus, lysosome, endosome, endoplasmic reticulum, nucleus, **plasma membrane**	Adaptive immune response, regulation of protein phosphorylation, regulation of cytokine‐mediated signaling pathway
RBPMS	3	RNA binding protein with multiple splicing	RBP‐MS, HERMES	Nucleus, cytosol	RNA processing, response to oxidative stress
SLC66A2	3	Solute Carrier Family 66 Member 2	PQLC1, FLJ22378	Golgi apparatus, endosome, **plasma membrane**	Retrograde transport, phospholipid translocation
ACTN4	2	Actinin alpha 4	FSGS1 3, ACTININ‐4 3, FSGS	Cytosol, nucleus, extracellular space	Actin cytoskeleton organization, protein transport
EMC10	2	ER membrane protein complex subunit 10	C19orf63, INM02, HSS1, HSM1	Endoplasmic reticulum, extracellular space	Angiogenesis, regulation of endothelial cell proliferation and migration
MLXIPL	2	MLX interacting protein‐like	WS‐BHLH, BHLHd14, MIO, WBSCR14, CHREBP, MONDOB	Nucleus	Regulation of transcription by RNA polymerase II
NDUFAF3	2	NADH:Ubiquinone Oxidoreductase Complex Assembly Factor 3	C3orf60, E3‐3, 2P1, MC1DN18	Mitochondrial inner membrane, nucleus	Mitochondrial respiratory chain complex I assembly
RANGAP1	2	Ran GTPase activating protein 1	KIAA1835, Fug1, SD	Cytosol, nucleus	Signal transduction, nucleocytoplasmic transport
ZFP36	2	Zinc finger protein 36	RNF162A, NUP475, G0S24, TTP, TIS11	Cytosol, nucleus	Regulation of transcription by RNA polymerase II, MAPK signal transduction
SCRN2	2	Secernin 2	SES2	Extracellular exosome	Proteolysis
AIP	1	Aryl hydrocarbon receptor interacting protein	XAP2, PITA1, ARA9, FKBP16, FKBP37	Nucleus, cytosol	Regulation of transcription, xenobiotic metabolic process, protein kinase A signaling
AKAP1	1	A kinase anchoring protein 1	PPP1R43, PRKA1, TDRD17	Mitochondrion, cytosol, **plasma membrane**	Apoptotic process, antiviral innate immune response
ARID5A	1	AT‐rich interaction domain 5A	MRF1, RP11‐363D14	Nucleus	Regulation of transcription by RNA polymerase II
ATAD3B	1	ATPase family AAA domain containing 3B	TOB3	Mitochondrion, **plasma membrane**	Mitochondrion organization
CCDC3	1	Coiled‐coil domain containing 3		Endoplasmic reticulum, extracellular	Regulation of tumor necrosis factor‐mediated signaling pathway, regulation of gene expression
CD24	1	CD24 molecule	CD24A	**Plasma membrane**	Regulation of cytokine‐mediated signaling pathway, response to hypoxia
COG1	1	Component of oligomeric Golgi complex 1	KIAA1381, LDLB	GOLGI apparatus	Protein transport, Golgi retrograde transport
DAZAP2	1	DAZ‐associated protein 2	KIAA0058, PRTB	Nucleus	Regulation of protein serine/threonine kinase activity
EBPL	1	Emopamil‐binding protein‐like	ERP, EBRP	Endoplasmic reticulum	Sterol metabolic process
FGFR1	1	Fibroblast growth factor receptor 1	BFGFR, CEK, FLG, N‐SAM, CD331, FLT2, KAL2, ECCL	**Plasma membrane**, cytosol, nucleus, extracellular region	MAPK cascade, epithelial to mesenchymal transition, protein phosphorylation
FTL	1	Ferritin light chain	NBIA3, LFTD, MGC71996	Lysosome, cytosol, extracellular region	Iron ion homeostasis, iron ion transport
HEXA	1	Hexosaminidase subunit alpha	TSD	Lysosome, cytosol, extracellular	Carbohydrate metabolic process, glycosaminoglycan biosynthetic process
HMGA1	1	High mobility group AT‐hook 1	HMGIY, HMGA1A, HMG‐R	Nucleus, cytosol	Nucleosome disassembly, base‐excision repair
HSPG2	1	Heparan sulfate proteoglycan 2	PRCAN, HSPG, PLC, SJA, SJS, SJS1	Extracellular, Golgi apparatus, lysosome, **plasma membrane**	Receptor‐mediated endocytosis, angiogenesis, lipid metabolic process, tissue development
IFFO1	1	Intermediate filament family orphan 1	HOM‐TES‐103, IFFO	Nucleus, cytosol, cytoskeleton	Double‐strand break repair via non‐homologous end joining
IGHA1	1	Immunoglobulin heavy constant alpha‐1	IgA1	**Plasma membrane**, extracellular region	Adaptive immune response
ITGB4	1	Integrin subunit beta 4	CD104, GP150	Extracellular, **plasma membrane**, nucleus	Cell communication, autophagy, cell adhesion, integrin‐mediated signaling pathway
JAM3	1	Junctional adhesion molecule 3	JAMC	**Plasma membrane**, extracellular, Golgi apparatus, cytoskeleton	Angiogenesis, adaptive immune response
KIT	1	KIT Proto‐Oncogene, Receptor Tyrosine Kinase	CD117, PBT, c‐Kit, SCFR, MASTC	Nucleus, extracellular, **plasma membrane**, cytosol	Cell differentiation, cell migration, signal transduction, regulation of MAPK cascade, regulation of programmed cell death, Kit signaling pathway
KLHL21	1	Kelch‐like family member 21	KIAA0469	Cytoskeleton, cytosol	Protein ubiquitination, regulation of cytokinesis
LENG8	1	Leukocyte receptor cluster member 8	KIAA1932, Pp13842	Nucleus, cytosol	‐
LMNA	1	Lamin‐A/C	LGMD1B, CMD1A, LMNL1, PRO1, LMNC, LFP, EMD2	Nucleus, nuclear envelope, cytosol, cytoskeleton	Protein import into nucleus, nucleus organization
LRP1	1	Low density lipoprotein receptor‐related protein 1	APOER, Alpha‐2‐Macroglobulin Receptor, IGFBP3R1, IGFBP‐3R, CD91	**Plasma membrane**, Golgi apparatus, lysosomal membrane, nucleus, cytoplasm	Receptor‐mediated endocytosis, lipid metabolic process
NFIC	1	Nuclear factor I/C	CTF, CTFS, NF‐I, NFI	Nucleus	Regulation of transcription by RNA polymerase II
NOTCH2	1	Notch receptor 2	HN2, AGS2, HJCYS	Nucleus, extracellular, **plasma membrane**	Notch signaling pathway, regulation of transcription by RNA polymerase II
NOTCH3	1	Notch receptor 3	CADASIL1, IMF2, LMNS, CASIL	Cytosol, nucleus, cytoskeleton, **plasma membrane**	Notch signaling pathway, regulation of transcription by RNA polymerase II
NAA60	1	N‐alpha‐acetyltransferase 60, NatF catalytic subunit	NAT15, HAT4, HNaa60	Golgi apparatus, cytoskeleton	Chromatin organization, N‐terminal protein amino acid acetylation
PAPLN	1	Papilin, Proteoglycan like sulfated glycoprotein	MGC50452, PPN	Extracellular region	Proteolysis, extracellular matrix organization, regulation of endopeptidase activity
PFKL	1	phosphofructokinase, liver type	ATP‐PFK, PFK‐B, PFK‐L	Cytoplasm, extracellular region	Glycolytic process, carbohydrate metabolic process
POLR2G	1	RNA Polymerase II Subunit G	RPB7, HRPB19, HsRPB7, RPB19	Nucleus	Transcription by RNA polymerase II
PRMT7	1	Protein arginine methyltransferase 7	SBIDDS	Cytosol, nucleus	Chromatin organization, spliceosomal snRNP assembly, protein methylation
PTGES2	1	prostaglandin E synthase 2	PGES2, MPGES2, GBF1	Golgi apparatus, cytosol, mitochondrion, lysosome, extracellular	Lipid metabolic process, prostaglandin biosynthetic process
RABGAP1	1	RAB GTPase activating protein 1	Rab6, TBC1D1, GAPCENA	Cytosol, cytoskeleton	Intracellular protein transport, regulation of GTPase activity
RBFOX1	1	RNA binding protein Fox‐1 homolog 1	HRNBP1, A2BP1, FOX‐1, 2BP1	Nucleus, Golgi apparatus	RNA splicing
SF1	1	Splicing factor 1	ZNF162, ZFM1, BBP	Nucleus	RNA splicing
TGFB1I1	1	Transforming growth factor beta 1 induced transcript	ARA55, HIC5, TSC‐5	Nucleus, cytosol	Cell adhesion, epithelial to mesenchymal transition
TNS1	1	Tensin 1	TNS, MXRA6, PPP1R155	Nucleus, cytoskeleton	Cell‐substrate junction assembly, intracellular signal transduction
TP53I3	1	Tumor protein p53 inducible protein 3	PIG3	Cytosol	NADP metabolic process
TSPO	1	Translocator protein	PKBS, PBR, MBR, MDRC, Pk18, BZRP, DBI, IBP	Cytosol, mitochondrion, extracellular, endoplasmic reticulum	Steroid biosynthetic process, protein targeting to mitochondrion
TXNIP	1	Thioredoxin‐interacting protein	THIF, VDUP1, ARRDC6, HHCPA78	Cytosol, nucleus, mitochondrion	Regulation of transcription by RNA polymerase II, response to oxidative stress, cell cycle
USP54	1	Ubiquitin specific peptidase 54	BA137L10.4, C10orf29	Mitochondrion	Protein deubiquitination
WBP2	1	WW domain binding protein 2	GRAMD6	Nucleus, cytosol	Epigenetic maintenance of chromatin in transcription‐competent conformation, regulation of intracellular estrogen receptor signaling pathway
WSB1	1	WD repeat and SOCS box containing 1	SWIP1	Cytosol	Protein ubiquitination, intracellular signal transduction

Cell membrane localizations are highlighted in bold.

^a^ Subcellular localization database COMPARTMENTS.

^b^ Gene Ontology database AmiGO 2.

### 3.2 CD74 interacts with TIMP‐1 in breast cancer cells

MHC class II‐associated invariant chain peptide (CD74) was identified in three of the original 78 positive clones, where one clone contained isoform a, and two clones contained isoform b of the CD74 protein (Table 1). CD74 is a major histocompatibility (MHC) class II chaperone [23]. Under physiological conditions, CD74 is expressed mainly in professional antigen‐presenting cells (APC) of the immune system. Several studies have demonstrated that CD74 is overexpressed in B‐cell neoplasms [24], and in solid tumors, including breast cancer [25]. CD74 was shown to interact with TIMP‐1 in human B cells [22], but under physiological conditions, CD74 is expressed at high levels by APC, including B cells, but not in breast epithelial cells [26]. CD74 is thought to promote breast cancer metastasis, so we decided to focus our attention on its putative interaction with TIMP‐1 in breast cancer cells.

The Genes‐to‐Systems Breast Cancer Database, a resource that integrates data about genes, transcripts, and proteins altered in breast cancer, shows a ratio value of CD63 expression for breast ductal carcinomas compared to normal tissue of 0.2281 [27]. Data from other available resources such as Cancer Genome Atlas, or Protein Atlas, supported the notion that expression of CD63 in breast tumors is uncommon. Given that too high, or too low, endogenous cellular levels of TIMP‐1 could confound our analyses, and that presence of CD63 would obscure the interpretation of experiments aiming to ascertain a role for CD74 in internalization of TIMP‐1, we chose as model system a breast cancer cell line with moderate‐to‐high expression of CD74, with concomitant moderate expression of TIMP‐1, and undetectable expression of CD63. Analysis of the expression levels of CD74, CD63, and TIMP‐1, respectively, in various breast cancer cell lines, including MDA‐MB‐231, MCF‐7, T47D, and SK‐BR‐3 (Fig. S1), identified MDA‐MB‐231 (high CD74; undetectable CD63; moderate TIMP‐1 expression) as the most adequate model system for us, and we used this line for all subsequent studies. We abrogated CD74 expression in MDA‐MB‐231 cells with a CD74‐specific siRNA (siRNA‐CD74) and compared the levels of TIMP‐1 in these cells to those of control siRNA‐transfected MDA‐MB‐231 cells (siRNAcontrol).

**Fig. 1:**
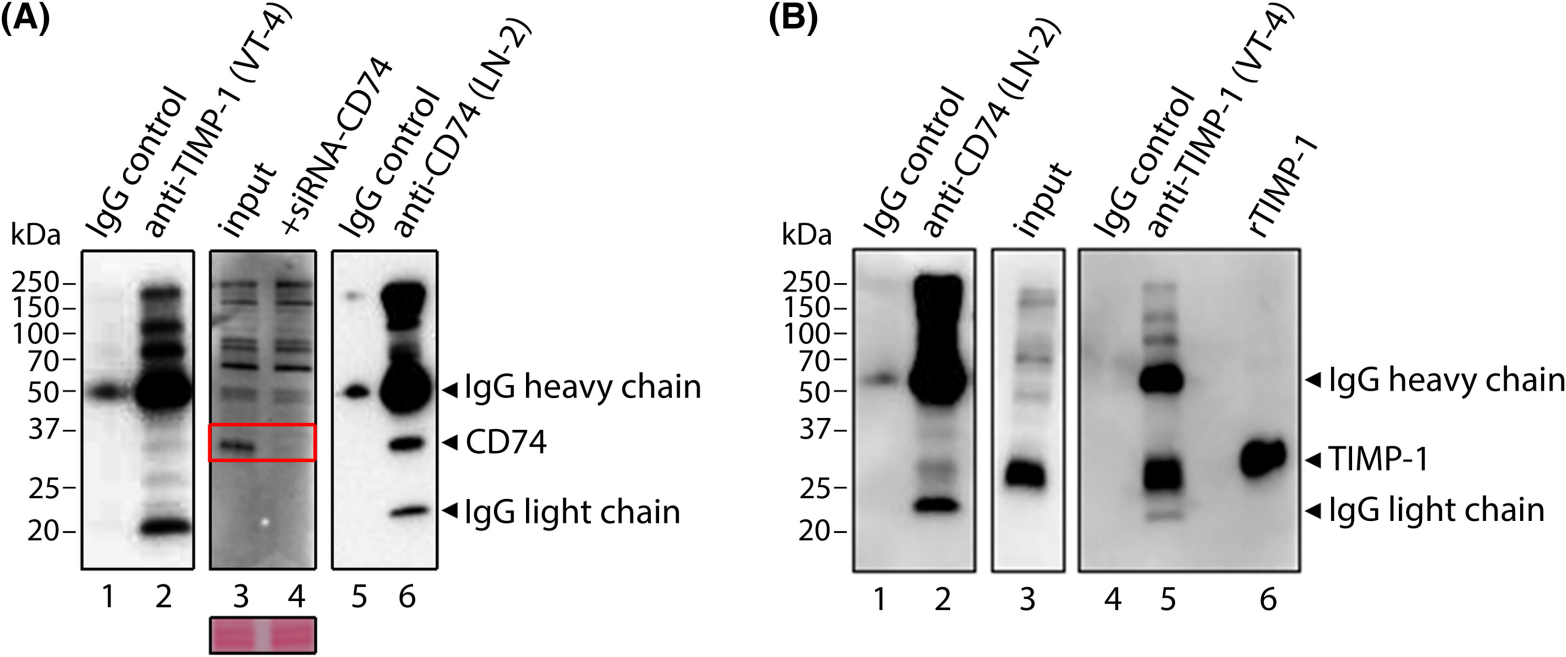
Protein interaction analysis of TIMP‐1 and CD74 in MDA‐MB‐231 breast cancer cells. Cultured cells were incubated with rTIMP‐1 (5 μg·mL^−1^) for 15 min before co‐immunoprecipitation. (A) Presence of CD74 protein in immunoprecipitates from MDA‐MB‐231 cell lysates using either an anti‐CD74 antibody (LN‐2 clone, rightmost panel) or an anti‐TIMP‐1 antibody (VT‐4 clone, leftmost panel), were analyzed by western blotting with an anti‐CD74 antibody (LN‐2 clone). (B) Presence of TIMP‐1 protein in immunoprecipitates from MDA‐MB‐231 cell lysates using either an anti‐CD74 antibody (LN‐2 clone, leftmost panel) or an anti‐TIMP‐1 antibody (VT‐4 clone, leftmost panel), were analyzed by western blotting with an anti‐TIMP‐1 antibody (VT‐7 clone). In all cases, control immunoprecipitations were performed in parallel using beads coated with IgG1 mouse immunoglobulins (A, lanes 1 and 5; and B, lanes 1 and 4, respectively). The input lanes (A and B, lane 3) were loaded with 5% of the corresponding MDA‐MB‐231 lysate. Arrows indicate the bands corresponding to non‐specific detection of IgG‐light chain (IgG‐LC) and IgG‐heavy chain (IgG‐HC) by the secondary antibody. The positions of the CD74 and TIMP‐1 bands were verified by a control sample with down regulation of CD74 by CD74 siRNA (A, lane 4), and loading of rTIMP‐1 (B, lane 6). Equal loading was confirmed by reversibly staining membranes with Ponceau S (illustrated with region of membrane including CD74; red rectangle). Results are representative of three independent experiments.

To confirm that a physical interaction between TIMP‐1 and CD74 does take place in breast cancer cells, we performed co‐immunoprecipitation studies using MDA‐MB‐231 breast cancer cells cultured in the presence of rTIMP‐1. Pull‐down of CD74 from MDA‐MB‐231 cell lysates was confirmed by western blotting with an anti‐CD74 antibody (Fig. 1B, lane 6). Co‐immunoprecipitated TIMP‐1 was visualized by western blotting using an anti‐TIMP‐1 antibody (VT‐7 clone). A band, corresponding in size to rTIMP‐1 control (Fig. 1A, lane 6) and endogenous TIMP‐1 in whole‐cell lysate (Fig. 1B, lane 3), suggested an interaction of the exogenously added rTIMP‐1 and CD74 in MDA‐MB‐231 cells (Fig. 1B, lane 2). At the same time, no band corresponding to either TIMP‐1 (Fig. 1B, lanes 1 and 4, respectively) or CD74 (Fig. 1B, lanes 1 and 5, respectively) was detected in the control sample using IgG1 coupled beads. We also performed the converse immunoprecipitation and western blotting analysis, immunoprecipitating TIMP‐1 with anti‐TIMP‐1 antibody (VT‐4) coupled beads and detecting co‐immunoprecipitated CD74 by western blotting using LN‐2 anti‐CD74 antibody. A band matching the size of CD74 detected in MDA‐MB‐231 whole‐cell lysate was visualized in the immunoprecipitated TIMP‐1 sample (Fig. 1A, lane 2). We confirmed that the band in question was indeed CD74, using whole‐cell lysate of MDA‐MB‐231 cells transfected with a CD74‐specific siRNA as control. The band visualized in the IP‐TIMP‐1 sample and in MDA‐MB‐231 whole‐cell lysate was nondetectable in the CD74 downregulated cells, confirming that this band corresponded to CD74 (Fig. 1A, compare lanes 3 and 4). Again, no bands were detected in the control sample using IgG1 coupled beads (Fig. 1A, lane 1 and Fig. 1B, lane 4). Efficient pull‐down of TIMP‐1 was confirmed by anti‐TIMP‐1 western blot (Fig. 1B, lane 5).

**Fig. 2:**
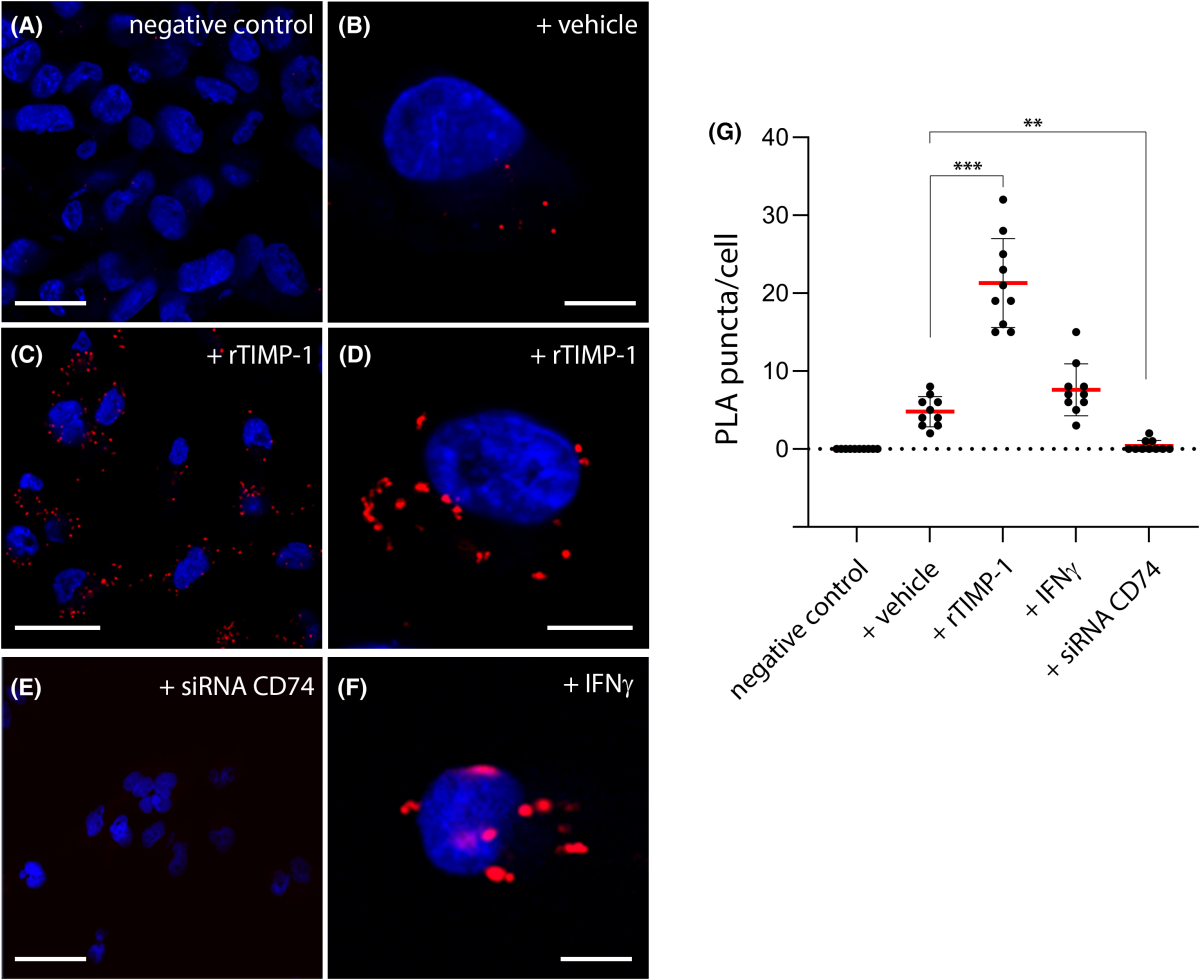
*In situ* proximity ligation assay (PLA) analysis of protein complexes consisting of CD74 and TIMP‐1. (A–F) The *in situ* PLA signal (red puncta) requires that CD74 and TIMP‐1 are in close proximity of each other (within 40 nm). (A) PLA signal was undetectable in negative control experiments, where only one antibody was incubated with the PLA probes. Anti‐CD74 was omitted in the shown experiment. (B) Under standard growth conditions, we found multiple PLA signals (red puncta). (C, D) Addition of rTIMP‐1 (5 μg·mL^−1^) drastically increased the number of visible PLA puncta. (E) Downregulation of CD74 with siRNA completely abolished the PLA signals. (F) Increasing expression of CD74 with IFNγ also resulted in increased number of PLA puncta. (G) Ten different regions were selected, and PLA puncta counted and normalized to cell number in each region. Results were plotted for each condition and presented as mean ± SD. Nuclei stained with DAPI dye (blue) are shown. Images were acquired across multiple *z*‐planes and combined. Representative images are shown for each condition. Data are representative of three independent experiments. Scale bars: (A, C, E)– 50 μm; (B, D, F)– 10 μm. ** denotes *P* < 0.01, and *** denotes *P* < 0.001. One‐way ANOVA was performed to compare the PLA puncta in MDA‐MB‐231 cells under the various conditions.

Having obtained biochemical evidence of an interaction between CD74 and TIMP‐1, we tried to corroborate the formation of CD74/TIMP‐1 complexes in a cellular context. We used *in situ* proximity ligation assay (PLA), a method that allows one to visualize protein–protein interactions in fixed cells, providing positional information [28]. Although direct binding between proteins cannot be proven through *in situ* PLA, the method allows the reliable *in situ* detection of two proteins in close juxtaposition (< 40 nm). We found multiple PLA signals in MDA‐MB‐231 cells, indicative of CD74/TIMP‐1 interactions (Fig. 2B). When exogenous rTIMP‐1 was added (Fig. 2C,D), the number of PLA signals increased significantly (Fig. 2G; *P* < 0.001). A similar increase in PLA signals, albeit of lesser magnitude, was also observed upon stimulation of MDA‐MB‐231 cells with IFNγ, which induces expression of CD74 (Fig. 2F,G). Conversely, abrogation of CD74 expression with siRNA‐CD74 eliminated the PLA signal (Fig. 2E; *P* < 0.01).

### 3.3 TIMP‐1 internalization and associated activation of Akt is CD74‐dependent

**Fig. 3:**
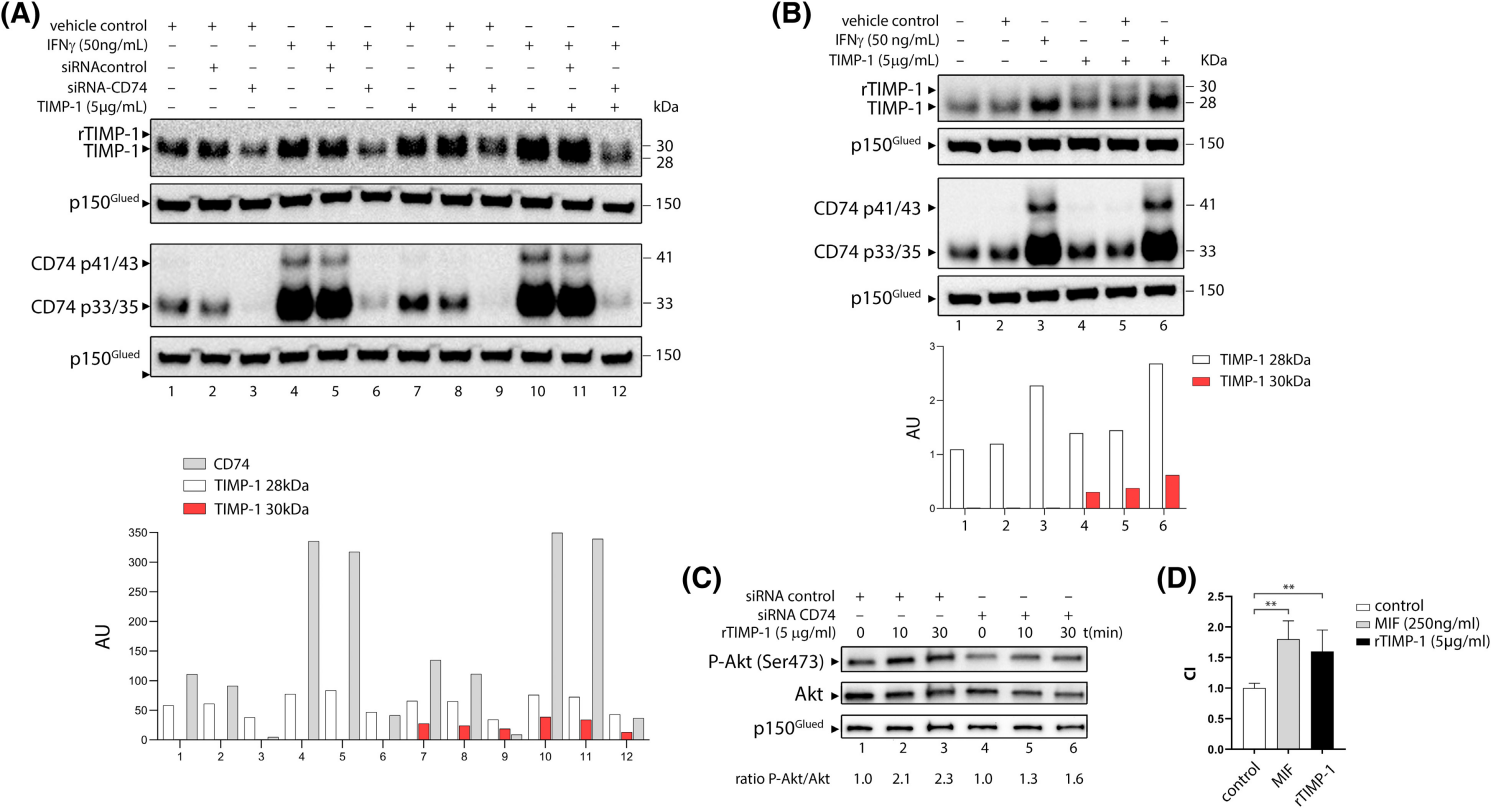
Expression of CD74 and TIMP‐1 in MDA‐MB‐231 cells. (A) To determine if CD74 was required for TIMP‐1 cellular internalization, MDA‐MB‐231 cells were transfected with siRNA‐CD74 or a negative control, siRNAcontrol. Alternatively, CD74 expression was stimulated with IFNγ. Subsequently, cells were cultured in the presence of 5 μg·mL^−1^ rTIMP‐1 or vehicle for 4 h. MDA‐MB‐231 cells transfected with a control siRNA showed no difference in CD74 expression compared to un‐transfected cells (lanes 1 and 2, respectively), whereas cells transfected with a CD74 specific siRNA, displayed more than 90% downregulation in CD74 expression (lane 3). Treatment with IFNγ greatly increased expression of CD74 (lane 4), whereas combined transfection of MDA‐MB‐231 cells with a CD74 siRNA and IFNγ, resulted in weak expression of CD74 (lane 6). (B) In MDA‐MB‐231 cells, stimulated or not with IFNγ, exposure to rTIMP‐1 for 30 min, showed the presence of this protein in cells in a CD74 dependent manner. (C) TIMP‐1 mediated Akt activation in MDA‐MB‐231 cells is dependent on CD74. Cells transfected with either control siRNA or a CD74 specific siRNA were, 72 h post transfection, exposed to rTIMP‐1 (5 μg·mL^−1^) for 10 or 30 min. Cell lysates, 20 μg per lane, were subjected to western blot analysis with antibodies against total Akt and P‐Akt (Ser473). Band signal intensities were measured using Image J at default settings for gel analysis. Band signals normalized to p150 loading controls are provided in the graphics below western blot images. Ratios between the signals from total Akt to pAkt, generated by normalizing band intensities to the signal ratio of control siRNA or CD74 siRNA cells at T0, respectively, are given. Identical protein loading was confirmed by reversibly staining blots with Ponceau S. The results shown are representative of three independent experiments. (D) TIMP‐1 showed a chemotactic effect on Ramos Burkitt's lymphoma cells. Cultured cells were subjected to transwell chemotaxis assay in response to MIF (250 ng·mL^−1^), or TIMP‐1 (5 μg·mL^−1^). Results are presented as a chemotaxis index (CI), with values normalized to spontaneous migration of B cells in medium in the absence of the chemoattractant. Data are expressed as CI (mean ± SD). ** denotes *P* < 0.01. Data are representative of three independent experiments. Unpaired *t*‐test was used to compare results from western blot analysis.

To investigate whether interaction with CD74 at the membrane was involved in the transport of TIMP‐1 across the plasma membrane, we examined the intracellular protein levels of TIMP‐1 in MDA‐MB‐231 cells, under standard growth conditions (Fig. 3A; lanes 1 and 2), upon stimulation with IFNγ (Fig. 3A; lanes 4 and 5), and in cells depleted for CD74 (Fig. 3A; lanes 3 and 6). Fig. 3A shows an illustrative experiment of the level of downregulation of CD74 expression with siRNA, and upregulation with IFNγ, respectively. As can be seen, CD74 is efficiently downregulated by the CD74 targeting siRNA (> 90% downregulation; lanes 3, 6, 9, and 12), whereas the negative control siRNA had no noticeable effect on CD74 expression (Fig. 3A, lanes 2, 5, 8, and 11). Conversely, IFNγ significantly increased the expression of CD74 (Fig. 3A, lanes 4, 5, 10, and 11). Depletion of CD74 resulted in decreased levels of TIMP‐1 (Fig. 3A, compare lane 3 with lane 2; *P* < 0.01). Conversely, upregulation of CD74 resulted in increased levels of TIMP‐1 (Fig. 3A, compare lanes 4 and 1; *P* = 0.03). Concomitant depletion of CD74 with siRNA and stimulation with IFNγ did not significantly change the protein levels of TIMP‐1 compared with just depletion of CD74 (Fig. 3A, compare lanes 4 and 1; *P* = 0.03), indicating that IFNγ is not directly affecting expression of TIMP‐1. To discriminate between an effect on expression of TIMP‐1 and uptake of extracellular TIMP‐1, we added exogenous rTIMP‐1 (5 μg·mL^−1^ for 4 h) to the culture medium and analyzed the levels of TIMP‐1. The recombinant TIMP‐1 protein we used is bioactive but has a polyhistidine affinity tag and is extensively glycosylated making it migrate in SDS/PAGE with an apparent molecular weight of 30 kDa, whereas the endogenous protein migrates with an apparent molecular weight of 28 kDa (Fig. 3A). Quantification of the two bands for each condition confirmed that depletion of CD74 also resulted in decreased levels of TIMP‐1 28 and 30 kDa (Fig. 3A, compare lane 9 with lane 8; *P* < 0.01). Conversely, upregulation of CD74 resulted in increased levels of TIMP‐1 28 and 30 kDa (Fig. 3A, compare lanes 10 and 7; *P* < 0.01). In all cases, cells transfected with control siRNA showed levels of TIMP‐1 comparable to those of nontransfected cells, indicating that siRNA transfection does not affect the uptake ability of the cells (Fig. 3A, compare lanes 1 and 2, lanes 4 and 5, lanes 8 and 7, and lanes 11 and 10, respectively).

A small fraction of the total CD74 protein expressed in cells is modified by the addition of chondroitin sulfate glycosaminoglycan, and this modified form of CD74 is expressed on the cell surface, where it can function as a receptor that is able to elicit various signaling events [29, 30, 31, 32]. Several studies have demonstrated that TIMP‐1 can modulate cell proliferation and cell survival through Akt phosphorylation [33, 34]. Likewise, signaling through CD74 has in several studies been shown to induce Akt phosphorylation [24, 35]. Thus, we wanted to investigate whether TIMP‐1 was able to stimulate phosphorylation of Akt in MDA‐MB‐231 cells and whether this effect was dependent on CD74. Given that signaling events are usually limited in time, we examined levels of TIMP‐1 after 30 min of exogenous rTIMP‐1 (Fig. 3B). As can be seen, the addition of rTIMP‐1 resulted in increased levels of TIMP‐1 (Fig. 3B, compare lanes 4 and 1, and lanes 5 and 2, respectively). Induction of CD74 with IFNγ resulted in increased levels of TIMP‐1 and comparatively higher levels of TIMP‐1 30 kDa compared to 28 kDa (Fig. 3B, compare lanes 6 and 5; *P* < 0.05).

We then determined phosphorylation levels of P‐Akt (Ser473) in MDA‐MB‐231 cells following exposure of these cells to rTIMP‐1 for 10 and 30 min, under normal conditions and in cells depleted for CD74. As shown in Fig. 3C, we observed an increase in P‐Akt (Ser473) following the addition of rTIMP‐1. Quantification of band intensities showed a P‐Akt/Akt ratio of 2.3 after 10 min‐ and 2.1 after 30 min of rTIMP‐1 exposure when normalized to P‐Akt/Akt ratio of the 0 min sample (Fig. 3C, compare lanes 3, 2 and 1, respectively). In contrast, CD74‐depleted cells displayed a markedly lower increase in P‐Akt/Akt ratio following rTIMP‐1 exposure when normalized to the control sample (Fig. 3C, *t* = 0 min). Thus, downregulation of CD74 resulted in a significant reduction in potential of Akt activation (Wilcoxon matched‐pair signed‐rank test; *P* = 0.03), showing an effect of CD74 on TIMP‐1‐mediated Akt signaling in MDA‐MB‐231 cells. But the CD74/TIMP‐1 interaction may also affect CD74 cellular functions. The MIF‐CD74 axis is a signaling pathway able to regulate B‐cell chemotaxis [36]. Given that TIMP‐1 interacts with CD74, we investigated whether TIMP‐1 itself can trigger B‐cell chemotaxis. The migratory capacity of Ramos B cells in response to MIF, TIMP‐1, or controls was assayed using a transwell migration assay. As shown in Fig. 3D, there was a chemotactic response toward MIF, as expected. But we also observed TIMP‐1‐induced B cell migration, although at a lower level than for MIF (Fig.3D).

### 3.4 Association between CD74, CD63, and TIMP‐1 expression in breast cancer

**Fig. 4:**
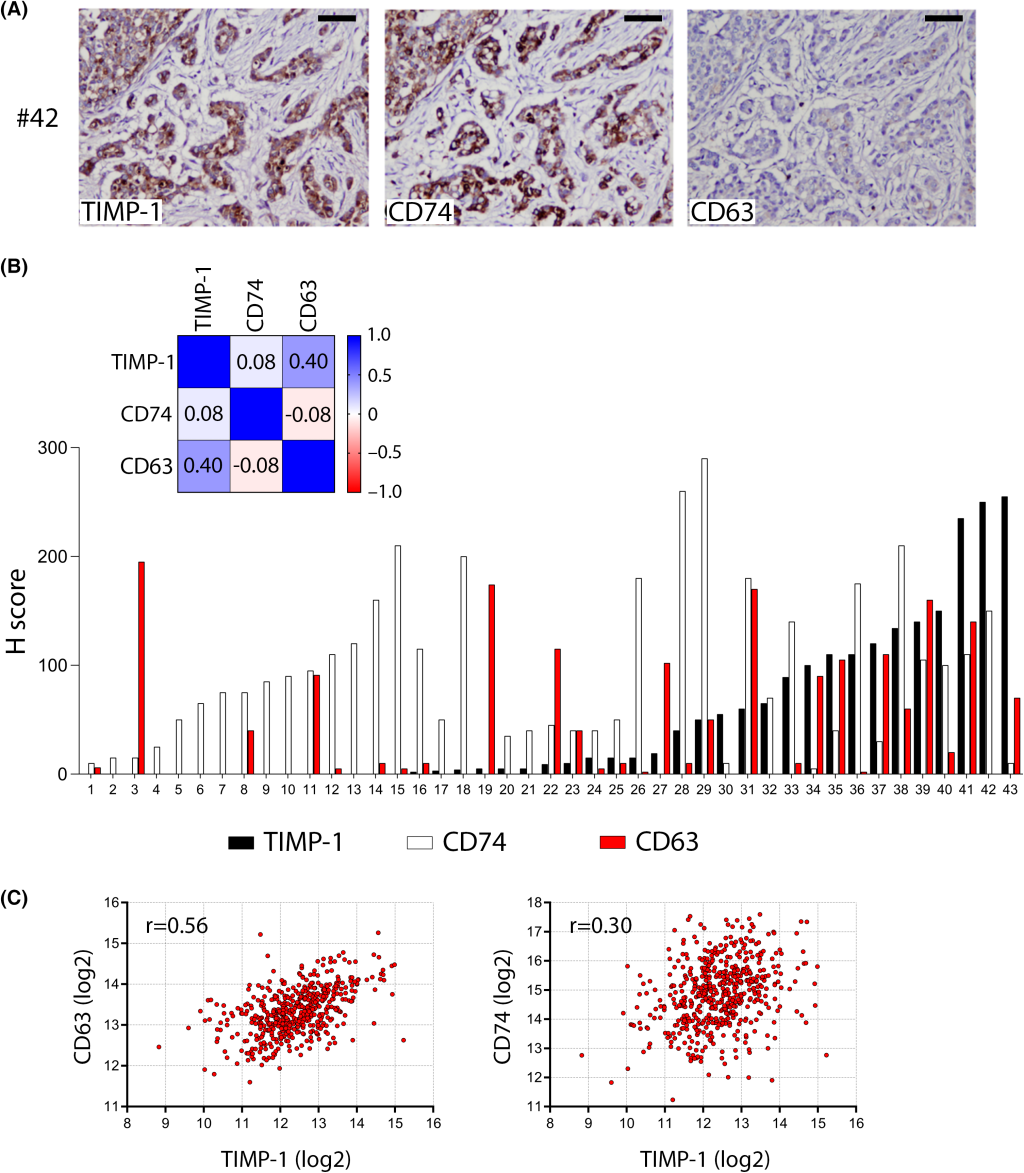
Association between expression of TIMP‐1, CD74, and CD63 in human breast tumor tissue. Serial sections of breast cancer samples were evaluated in parallel by (A) IHC staining of TIMP‐1 protein using a specific anti‐TIMP‐1 antibody (VT‐7 clone), IHC detection of CD74 protein, and IHC staining of CD63 expression. Original magnification, 20×. Scale bars, 50 μm. (B) Expression levels of TIMP‐1, CD74 and CD63 as evaluated by H‐scoring of 43 immunostained breast tissue samples. Samples are plotted after their TIMP‐1 H‐score (low to high). Matrix Spearman correlation factors were calculated for each case to examine the association between CD63, CD74, and TIMP‐1 H scores, and are presented in the figure inset. (C) Correlation analysis of *CD74*, *CD63*, and *TIMP‐1*. Gene expression levels for these genes was retrieved from GDAC Firehose gene expression dataset for TCGA Breast Invasive Carcinoma mRNA (RNA Seq) and plotted. Spearman correlation factors were calculated for each tested association.

Our group has previously reported the immunoreactivity pattern of TIMP‐1 protein in breast cancer cells in tissue samples [37]. We have also shown that expression of CD63 and TIMP‐1 appears to be correlated with glioblastoma [38]. To determine whether CD74 expression correlated with that of TIMP‐1 breast cance, we examined TIMP‐1, CD74, and CD63 protein levels in tumor cells in a set of 43 breast cancer tumors. The H‐scores for the protein levels present in the tumor cells are shown in Fig. 4 and Table S1, with results sorted by increasing TIMP‐1 levels. CD74 expression was observed throughout tumors at varying levels with no apparent correlation to TIMP‐1 (Spearman *r* = 0.08), whereas CD63 showed a moderate association with TIMP‐1 (Spearman *r* = 0.40; *P* = 0.004). Several tumors showed a trend toward expressing either CD74 or CD63 suggesting that TIMP‐1 may utilize either one of them as a cellular transporter. Correlative analysis of CD63, CD74, and TIMP‐1 expression in a Breast Invasive Carcinoma (TCGA, Firehose Legacy) dataset of 1108 samples showed a moderate association between CD63 expression and TIMP‐1 (Fig.4C; lefthand panel, Spearman *r* = 0.56), whereas CD74 expression was not associated with TIMP‐1 (Fig.4C; middle panel, Spearman *r* = 0.30), supporting our own data.

## 4 Discussion

### 4.1 Identification of potential TIMP‐1 interactors

The identification of CD63 as a cell surface binding partner for TIMP‐1, able to regulate cell survival and polarization through TIMP‐1‐mediated modulation of the tetraspanin/integrin signaling complex, and independently of its MMP‐inhibitory function, prompted a flurry of research activity aiming to identify molecular mechanisms underlying the various cellular roles of TIMP‐1. Consequently, there has, within the last decade, been a renewed interest in the identification of TIMP‐1 interacting proteins. Not least because of inconsistent or even opposing findings related to TIMP‐1‐associated physiological or pathological effects. Several lines of evidence pointed towards the existence of multiple interacting proteins. We performed an Y2H screening of a mammary gland cDNA library and identified 56 unique potential TIMP‐1 interactors. These included two well‐known interactors, CD63 [9] and LRP1 [11], and two previously reported TIMP‐1 associated proteins, CD74 [21, 22] and c‐Kit [20]. Some of the hits we identified can be connected to known interactor networks of TIMP‐1, such as the tetraspanin/integrin network (Table 1; ITGB4, or KIT), whereas others are suggestive of hitherto unknown functions of TIMP‐1, such as RNA splicing (Table 1; SF1, RBPMS, and RBFOX1). Others yet, such as SCRN2, may be related to the anti‐proteolytic activity of TIMP‐1.

Grünwald et al. [14] argued recently in an elegant and coherent overview of the relevant literature that the multiple functions of TIMP‐1 are interrelated in a biologically meaningful way, cooperating to determine TIMP‐1‐driven physiological or pathological effects. One can glimpse some of these interrelationships in the interactors we identified. Case in point: the small GTPase related proteins we identified (Table 1; ARHGDIA, RABGAP1, or RANGAP1). A recent report identified the small GTPase Rab37 as a novel metastasis suppressor that controls TIMP1's exocytosis leading to inactivation of MMP9 signaling. This finding provided a link between membrane trafficking and regulation of MMP actvitity [39]. The Rho and Rab GTPase related proteins we identified may increase our insight into this process.

We reported here the identification of several potential interactors, but now one needs to systematically, and experimentally, examine whether these are bona fide binders. Even more, how these interactors may contribute to the cellular functions of TIMP‐1. But this is not a trivial matter, as context will most certainly affect how and whether these proteins interact with TIMP‐1. We could show that CD74 interacted with TIMP‐1 in pull‐down assays (Fig. 1), and *in vivo* PLA assays (Fig. 2), but we were unable to detect any molecular interactions in solution using microscale thermophoresis (MST) (Fig. S2), a powerful technique to study protein–protein interactions in solution [40].

### 4.2 CD74 as a TIMP‐1 binding protein in breast cancer cells

CD74, also known as MHC class II invariant chain (li), was originally thought to function as an MHC class II chaperone promoting the exit of MHC class II molecules from the endoplasmic reticulum (ER) where it also prevents peptide binding, directing them to endocytic compartments and contributing to peptide editing in the MHC class II compartment [23]. However, a small percentage of total CD74 protein has been found to traffic to the plasma membrane where it functions as an accessory‐signaling molecule [29]. The surface half‐life of CD74, in complex with class II α and β chains, has been measured to be < 10 min, as CD74 is quickly recycled back into the endosomal pathway [41, 42, 43]. This particular property of CD74 has been successfully used for targeted drug delivery to cancer cells [44]. Although CD74 was originally linked to MHC class II, growing evidence indicates that CD74 can have multiple additional functions. CD74 can interact with multiple co‐receptors with different outcomes, integrating signals from various downstream signaling pathways to gain cell‐ and context‐specificity. Under the conditions of our assays we found that CD74 interacted with TIMP‐1 (Figs 1 and 2), and that this interaction was important for cellular internalization of TIMP‐1 (Fig. 3).

Our data raised one important question: does intracellular uptake of TIMP‐1 by MDA‐MB‐231 cells drive cellular signaling or is signaling a consequence of TIMP‐1 binding to CD74 on the cell surface, and internalization is an epiphenomenon caused by recycling of membrane‐bound CD74 into the endosomal pathway? In the former scenario CD74 functions as a transporter, in the latter case it would be a receptor. CD74 and TIMP‐1 have independently been reported to be deeply involved in various cell signaling processes and it is plausible that interaction between these two proteins would lead not just to internalization of TIMP‐1 but actual signaling. CD74 can, through binding of its well characterized ligand, macrophage‐migration inhibitory factor (MIF) and CD44, induce intracellular signaling pathways involving Syk tyrosine kinase, PI3K and Akt which in turn regulate survival of B cells [24, 35]. Likewise, TIMP‐1 has been shown in fibroblasts to induce cell proliferation through activation of PI3K and subsequent phosphorylation of Akt [45]. We have also shown that in colorectal cancer cells, exogenously added TIMP‐1 inhibits c‐Kit shedding and activates the c‐Kit signaling axis [20]. Other studies have demonstrated an involvement of TIMP‐1 mediated activation of the Janus Kinase 2 (JAK2)/PI3K/Akt/Bcl‐2 survival pathway in suppression of apoptosis in UT7 erythroid cells through interaction with pro‐MMP‐9 and CD44 on the cell surface [33, 46]. We showed that exogenously added rTIMP‐1 initiates an immediate response involving the Akt signaling cascade in MDA‐MB‐231 cells, which was dependent upon CD74 expression (Fig. 4). Activation of Akt can lead to diverse cellular responses such as cell division, suppression of apoptosis, increasing cell size and regulation of autophagy [47, 48, 49] indicating that some of the effects ascribed to TIMP‐1 can, at least in part, be due to its interaction with CD74. But even assuming that a direct effect on signaling takes place upon binding of TIMP‐1 to CD74, this is unlikely to be a binary interaction, and one would expect another protein to be involved in any TIMP‐1/CD74 direct signaling event, as the short cytoplasmic domain of membrane bound CD74 makes it unsuitable for direct interaction and activation of signaling pathways. An obvious candidate for a third binding protein could be CD44, a single pass trans‐membrane protein with known kinase activation properties which previously has been described as co‐receptor for both TIMP‐1 and CD74 [46, 50, 51, 52]. The functional interplay between CD74 and TIMP‐1 is not limited to effects on TIMP‐1 mediated Akt signaling, as we found that TIMP‐1 could mediate B cell chemotaxis through CD74 (Fig. 3D).

Another issue raised by our data concerns the biological effect of concomitant TIMP‐1 and CD74 expression and their association in tumor cells. Although CD74 overexpression is mostly associated with hematologic malignancies, it has also been reported in non‐hematopoetic cancers such as gastric, renal, urinary bladder, non‐small cell lung cancer certain sarcomas and glioblastomas [53, 54, 55, 56, 57]. A few studies have also reported CD74 expression in breast cancer, especially associated to the triple negative subtype and breast cancers with lymph node metastasis [25, 58, 59, 60]. However, the biological function(s) of CD74 in tumor cells is largely unknown. In a recent study, CD74 was shown to shift the localization of the tumor suppressor Scribble as well as down‐regulate the expression of the protein in cancer cells [61]. This was suggested by the authors to influence the motility and invasiveness of cancer cells and could therefore be one explanation for the correlation between high CD74 expression in triple negative tumors and their heightened metastatic propensity. A large proportion (> 95%) of the 43 tumors analyzed in this study showed expression of CD74 (Fig. 4), compared to only 40% tumors with any detectable CD63 expression (17 out of 43), confirming previous reports of CD74 being expressed in breast cancer, but also indicating that the CD74/TIMP‐1 interaction may be the major TIMP‐1 associated signaling axis, at least in breast cancer. Breast cancer is a highly heterogeneous disease and our data cannot unequivocally tell us whether the TIMP‐1/CD74 interaction is of pathophysiological relevance regarding tumor progression or response to treatment. To do this, larger, specific studies are needed. However, this is beyond the scope of this paper. The functional association we observed between CD74 and TIMP‐1 may well extend beyond breast cancer and classical cellular matrix and cell–cell interactions and motility, as CD74 is involved in tissue homeostasis where MMPs and TIMP‐1 plays a critical role, such as protection against injury and healing [62, 63], and modulation of immune responses [64].

## 5 Conclusions

Briefly, we have identified multiple TIMP‐1 interactors and verified that CD74 is a novel TIMP‐1 cell surface binding protein in breast cancer cells. Furthermore, we showed that CD74 is involved in the uptake of rTIMP‐1 by the triple negative breast cancer cell line, MDA‐MB‐231, and that this binding effects Akt signaling, which can lead to various cell responses. The clinical data presented in this paper additionally suggests a role for CD74 in the internalization of TIMP‐1 *in vivo*. Taken together, these data give new insights into the complex nature of TIMP‐1 and they can aid in explaining so far unresolved functions of TIMP‐1 with specific emphasis on cancer progression. In addition, the functional cross‐talk between the MIF/CD74/CD44 and TIMP‐1/CD63/CD44 pathways may extend beyond simple fortuitous interactions, but a bona fide regulatory mechanism linking inflammation, and immune responses, to tissue remodeling. Upon tissue damage TIMPs and MMPs are released to reorganize and heal, through controlled reorganization of the extracellular matrix. This process is known to involve an inflammatory response, thought to occur primarily through proteolytic maturation of cytokines, but there is a growing body of evidence linking CD74 with tissue injury repair of various tissues [62]. Also there is a comparable growing awareness of links between TIMP‐1 and inflammation [6, 65]. This functional association is noteworthy in cancer, and in particular in relation to the metastatic process, which involves tissue remodeling and inflammation. Further studies are warranted to establish whether TIMP‐1 and CD74 function as tropism factors for cancer cells and whether targeting their interaction would result in a novel therapeutic treatment for patients.

## Conflict of interest

The authors declare no conflict of interest.

## Author contributions

MH, JBN, MVV, UL and JS participated in the experimental design, statistical analysis, and interpretation of results. MH, JBN, and MVV carried out the experiments. MH, JS, UL, EMB and ATF designed and carried out the two‐hybrid screening. NB, SL and JMAM designed and supervised the study. SBN and AB collected patients' information, performed experiments, and made statistical analysis. MH and JMAM drafted and revised the manuscript. NB, SL, ATF and JS critically revised the manuscript for important intellectual content. All authors read and approved the final manuscript.

## Supporting information


**Fig. S1.** Expression levels of Timp‐1, CD74, and CD63 in breast cancer cell lines.

**Fig. S2.** MST interaction analysis between TIMP‐1 and CD74.

**Table S1.** H‐score of TIMP‐1, CD63, and CD74 expression in breast cancer samples.

## Data Availability

The data that supports the findings of this study are available in Table 1 and the supplementary material of this article. The data analyzed in Fig. 4 were derived from the following resource available in the public domain: GDAC Data Portal, https://portal.gdc.cancer.gov/.
