# Supplementary figures and images for: The invariant chain CD74 protein is a cell surface binding partner of TIMP‐1 in breast cancer cells
Source: Mol Oncol. 2023 Apr 28;17(8):1595–612. doi: 10.1002/1878-0261.13436 (PMC10399710; doi:10.1002/1878-0261.13436)

**MDA-MB-231**

**T47D**

**MCF-7**

**SKBR3**

**Timp-1**

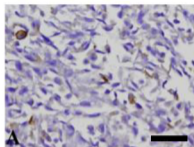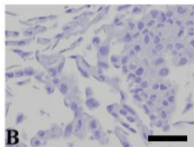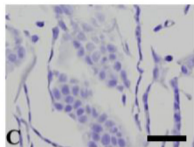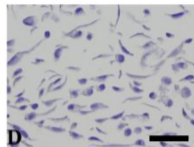

**CD74**

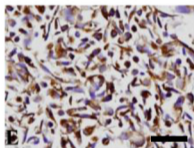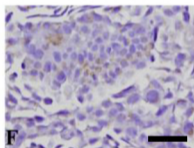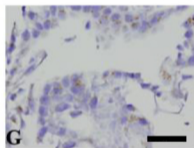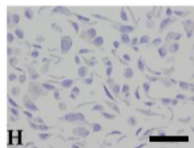

**CD63**

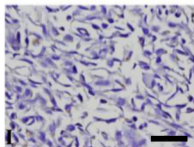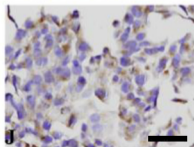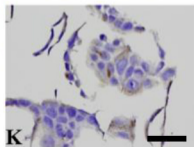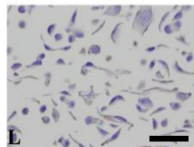

Supplement: Supplementary file 1 — Fig. S1. Expression levels of Timp‐1, CD74, and CD63 in breast cancer cell lines. [file MOL2-17-1595-s002.pdf]

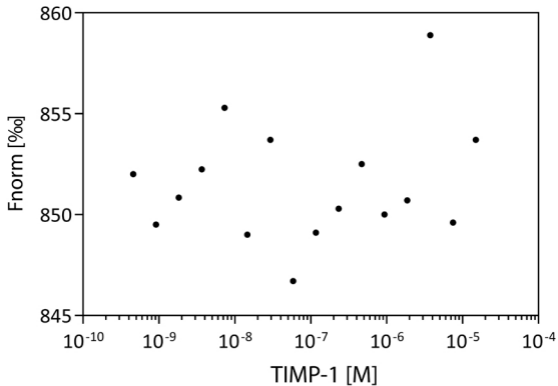

Supplement: Supplementary file 2 — Fig. S2. MST interaction analysis between TIMP‐1 and CD74. [file MOL2-17-1595-s003.pdf]
