# Supplementary material for: The invariant chain CD74 protein is a cell surface binding partner of TIMP‐1 in breast cancer cells
Source: Mol Oncol. 2023 Apr 28;17(8):1595–612. doi: 10.1002/1878-0261.13436 (PMC10399710; doi:10.1002/1878-0261.13436)
Supplement: Supplementary file 3 — Table S1. H‐score of TIMP‐1, CD63, and CD74 expression in breast cancer samples. [file MOL2-17-1595-s001.docx]

**Supplementary figure 1.** Expression levels of Timp-1, CD74 and CD63 in breast cancer cell lines. Expression of Timp-1, CD74 and CD63 in the four human breast cancer cell lines; MDA-MB-231, T47D, MCF-7 and SKBR3 was evaluated by IHC staining. **(a-d)** IHC staining of Timp-1 protein using a specific anti-TIMP-1 antibody (VT-7 clone), **(e-h)** IHC staining of CD74 expression using a specific anti-CD74 antibody (LN-2 clone) and **(i-l)** IHC staining of CD63 expression using a specific anti-CD63 antibody (NKI/C-3 clone). Original magnification, x 40. Scale bars, 50µm.

**Supplementary figure 2.** MST interaction analysis between TIMP-1 and CD74. MST values of a single representative experiment are shown. CD74-YFP containing cell lysate was prepared according to a protocol of the experimenter. In the MST experiment, we have kept the concentration of CD74-YFP containing cell lysate constant, while the concentration of the non-labeled TIMP-1 was varied between 0.45 nM – 15 µM. The assay was performed in PBS buffer, containing 0.05% Tween20 and 50% of cell lysate buffer. MST experiments were carried out at medium MST power at 25 °C. Prior to titration, TIMP1 was centrifuged down at 13.000 rpm for 10 minutes. The samples were loaded into MST NT.115 standard glass capillaries and the MST analysis was performed using the Monolith NT.115. Concentrations on the x-axis are plotted in M. Dose-response curves were fitted when possible to a one-site binding model to extract Kd values. Fnorm = normalized fluorescence.

**Supplementary table 1.** H-score of TIMP-1, CD63, and CD74 expression in breast cancer samples

|  | **IHC H-score** | | |
| --- | --- | --- | --- |
| **Patient #** | **TIMP-1** | **CD63** | **CD74** |
| 1 | 0 | 0 | 120 |
| 2 | 0 | 0 | 65 |
| 3 | 0 | 5 | 210 |
| 4 | 0 | 0 | 75 |
| 5 | 0 | 40 | 75 |
| 6 | 0 | 0 | 25 |
| 7 | 0 | 0 | 90 |
| 8 | 0 | 91 | 95 |
| 9 | 0 | 0 | 85 |
| 10 | 0 | 10 | 160 |
| 11 | 0 | 6 | 10 |
| 12 | 0 | 0 | 50 |
| 13 | 0 | 5 | 110 |
| 14 | 0 | 195 | 15 |
| 15 | 0 | 0 | 15 |
| 16 | 2 | 10 | 115 |
| 17 | 3 | 0 | 50 |
| 18 | 4 | 0 | 200 |
| 19 | 5 | 0 | 40 |
| 20 | 5 | 0 | 35 |
| 21 | 5 | 174 | 0 |
| 22 | 9 | 115 | 45 |
| 23 | 10 | 40 | 40 |
| 24 | 15 | 2 | 180 |
| 25 | 15 | 10 | 50 |
| 26 | 15 | 5 | 40 |
| 27 | 19 | 102 | 0 |
| 28 | 40 | 10 | 260 |
| 29 | 50 | 50 | 290 |
| 30 | 55 | 0 | 10 |
| 31 | 60 | 170 | 180 |
| 32 | 65 | 0 | 70 |
| 33 | 89 | 10 | 140 |
| 34 | 100 | 90 | 5 |
| 35 | 110 | 2 | 175 |
| 36 | 110 | 105 | 40 |
| 37 | 120 | 110 | 30 |
| 38 | 134 | 60 | 210 |
| 39 | 140 | 160 | 105 |
| 40 | 150 | 20 | 100 |
| 41 | 235 | 140 | 110 |
| 42 | 250 | 0 | 150 |
| 43 | 255 | 70 | 10 |
